# Mesenchymal stem cells enhance ovarian cancer cell infiltration through IL6 secretion in an amniochorionic membrane based 3D model

**DOI:** 10.1186/1479-5876-11-28

**Published:** 2013-01-31

**Authors:** Cyril Touboul, Raphael Lis, Halema Al Farsi, Christophe M Raynaud, Mohamed Warfa, Hamda Althawadi, Eliane Mery, Massoud Mirshahi, Arash Rafii

**Affiliations:** 1 Stem cell and microenvironment laboratory, Weill Cornell Medical College in Qatar, Education City, Qatar Foundation, Doha, Qatar; 2 UMRS 872 INSERM, Université Pierre et Marie Curie, Equipe 18, Centre de Recherche des Cordeliers, 15 rue de l’Ecole de Medecine, Paris Cedex 06, 75270, France; 3 Department Genetic Medicine, Weill Cornell Medical College, New York, USA; 4 Department of Pathology, Institut Claudius Regaud, Toulouse, France; 5 Department of Genetic Medicine and Obstetrics and Gynecology, Weill Cornell Medical College, Stem cell and microenvironment laboratory, Weill Cornell Medical College in Qatar, Qatar-Foundation, 24144, Doha, Qatar

**Keywords:** Ovarian cancer, IL6, Tumor infiltration, 3d model, Mesenchymal stem cell

## Abstract

**Background:**

The early peritoneal invasion of epithelial ovarian cancer (EOC) by tumoral aggregates presents in ascites is a major concern. The role of the microenvironment seems to be important in this process but the lack of adequate models to study cellular interactions between cancer cells and stromal cells does not allow to uncover the molecular pathways involved. Our goal was to study the interactions between ovarian cancer cells (OCC) and mesenchymal stem cells (MSC) using a 3D model.

**Methods:**

We used millimetric pieces of amniochorionic membrane - referred to as amniotic membrane scaffold (AMS) - to create 3D peritoneal nodules mimicking EOC early invasion. We were able to measure the distribution and the depth of infiltration using confocal microsopy. We extracted MSC from the amniochorionic membrane using the markers CD34-, CD45-, CD73+, CD90+, CD105+ and CD29+ at the Fluorescence Activated Cell Sorting (FACS) analysis. We used transwell and wound healing tests to test OCC migration and invasion in vitro.

**Results:**

Here we show that OCC tumors were located in regions rich in MSC (70%). The tumors infiltrated deeper within AMS in regions rich in MSC (p<0.001). In vitro tests revealed that higher IL6 secretion in a context of MSC-OCC co-culture could enhance migration and invasion of OCC. After IL6 receptor antagonism, OCC infiltration was significantly decreased, mostly in regions rich in MSCs, indicating that recruitment and tridimensional invasion of OCC was dependent of IL6 secretion.

**Conclusions:**

The use of tridimensional models using AMS could be a useful tool to decipher early molecular events in ovarian cancer metastasis. Cytokine inhibitors interrupting the cross-talk between OCCs and MSCs such as IL6 should be investigated as a new therapeutic approach in ovarian cancer.

## Background

Epithelial Ovarian carcinoma (EOC) is the sixth most common malignancy in woman and the leading cause of death from gynecological cancer [1,2]. One of the main differences between EOC and other neoplasm is burden of local extension. Indeed tumor cells spread leads rapidly to peritoneal carcinosis. Hence the majority of mortality in EOC is due to extensive peritoneal disease, with an overall survival ranging from 20 to 30% at 5 year [3].

While many studies in the literature address the issue of distant metastasis through blood circulation, the biology of peritoneal tumor spread in advanced EOC is not well known. Development of peritoneal carcinomatosis involves well-defined critical steps, including cells shedding and transport, interaction and adhesion to mesothelial layer, as well as colonization and proliferation into the sub-mesothelial microenvironment [4]. During the invasion of the sub-mesothelium ovarian cancer cells (OCC) interact with a complex stroma containing cells such as inflammatory cells and mesenchymal stem cells (MSC). A growing number of studies underlie the role of the microenvironnement in EOC peritoneal spreading. Bourguignon et al. enlightened the involvement of hyaluronan-CD44 (hyaluronan receptor) in early adhesion of OCC to peritoneal sheath [5]. We have previously demonstrated the role of MSC in ovarian tumor growth and resistance to therapy [6-8]. The lack of optimal models to mimic peritoneal extension is a limitation to decipher molecular events implicated in the interaction between cancer and stromal cells. Indeed most OCC exfoliate in the peritoneal cavity and evolve as tumoral aggregates. Hence, classical 2D cultures might not represent an ideal model to reproduce stepwise metastasis initiation [9].

The peritoneum is a complex organ composed by the mesothelium, a simple squamoid epithelium lining also the pleural and pericardial cavities. This surface epithelium is attached to a basement membrane lying on a stroma of variable thickness constituted by a collagen-based matrix, blood and lymphatic vessels, nerve fibers, and, in the normal state, rare hematogenous cells [10]. Two joint membranes compose the amniochorionic membrane (AM): the amnion and the chorion. The amnion is composed by a monolayer of epithelial cells separated from a mesenchymal cellular stroma by a thick basement membrane [11]. It has been used in various studies to mimick the peritoneum [12,13]. The rich content of MSCs in the amniochorionic membrane might also be an optimal tool to understand the interaction between cancers cells and stromal cells [11,14].

In this study, we hypothesized that MSCs could play a role in the infiltration of OCC in the sub-mesothelial layer. We demonstrated that AMS is an appropriate tool to study early adhesion of OCC aggregates to epithelial sheath and early invasion into sub-mesothelial layer. We were able to correlate the distribution of OCCs infiltration with the presence of MSC within the AMS. IL6 was found as a factor secreted in co-culture between MSC and OCC and was a determinant factor for OCC infiltration within the AMS.

## Material and methods

### Culture of ovarian cancer aggregates with amniochorionic membrane

We used OVCAR 3 and SKOV 3 cell lines previously transfected with eGFP label. Fifty thousand ovarian cancer cells (OCC) were cultivated in ultralow attachment 48 well plate (Corning) in DMEM/F12 (1:1) (Hyclone) basal media supplemented with 2 mM L-Glutamine (Hyclone), 1 × Non Essential Amino Acid (NEAA) (Hyclone), PenStrepAmpB (Sigma), 20 ng/ml basic Fibroblast Growth Factor (bFGF) (Peprotech), 20 ng/ml Epidermal Growth Factor (EGF) (Peprotech), 5 μg/ml Insulin (Sigma), 2% B27 supplements (Invitrogen) and 4% basement matrigel (BD Biosciences). Cultures were incubated in humidified 5% CO2 incubators and the media was replaced every 3 days.

Following approval form the Internal review Board (HMC- IRB protocol 9109/09, Weill Cornell Medical College in Qatar), placenta and amniochorionic membranes were collected from donors at Woman’s Hospital at Hamad Medical Corporation immediately after elective caesarean section in the absence of labor, preterm rupture of membrane-chorioamniontitis. The amniochorionic membrane was washed with PBS and red blood cells were removed using RBC lysis buffer (eBiosciences). Millimetric pieces of amniochorionic membrane (referred to as amniotic membrane scaffold (AMS)) were co-cultivated with the OCC.

### Fluorescence activated cell sorting (FACS) analysis

Amniochorionic membrane was washed with PBS and red cells were removed with RBC lysis buffer (eBiosciences). We then chunked and digested the membrane in a pre-warmed cocktail of Dispase 2 (1 mg/ml, Stem Cells Inc) and Collagenase/Hyaluronidase (300 μg/ml and 100 μg/ml respectively, Stem Cells Inc) during 45 minutes at 37°C. We then filtered the digested tissue in a 100 μm filter and obtained a cell suspension. Cell suspension was stained with Mouse anti-human CD45 antibody (BD Biosciences, #339192, clone 2D1) coupled with Amcyan, Mouse anti-human CD34 (BD Biosciences, #555821, clone 581) coupled with FITC, Mouse anti-human CD105 (biolegend, #323212, clone 43A3) coupled with AF647, Mouse anti-human CD73 (BD Biosciences, #550257, clone AD2) coupled with PE, Mouse anti-human CD29 (biolegend, #323212, clone TS2/16) coupled with APC-Cy7, Mouse anti-human CD90 (BD Biosciences, #550402, clone 5E10) coupled with AF700, Mouse anti-human CD44 (BD Biosciences, #555479, clone G44-26) coupled with PE.

Briefly, 1.10^6^ cells were harvested and non-specific binding prevented by blocking in PBS- 5% FBS-1% BSA-10% FcR Blocking Reagent (Myltenyi Biotec) for 30 minutes on ice. Cell suspension was incubated with specific antibodies for 45 minutes on ice. Filtered, single-cell suspension was analyzed by Fluorescence Activated Cell Sorting (FACS) on a SORP FACSAria2 (BD Biosciences). Data were processed with FACSDiva 6.3 software (BD Biosciences). Doublets were excluded by FSC-W × FSC-H and SSC-W × SSC-H analysis, single stained channels were used for compensation, and fluorophore minus one (FMO) controls were used for gating, 500 000 events were acquired per sample.

### Confocal analysis

Tumors were washed two times with PBS and fixed with paraformaldehyde 3.7% (Sigma) before staining. The antibodies used were the mouse anti human CD73_APC (Biolegend, #344006, clone AD2) and CD90_PerCP-Cy5.5 (Biolegend, #328118, clone 5E10). The non-specific sites were blocked with PBS with 0.3% bovine serum albumin and 0.5% HS during one hour. Tumors were incubated with the antibodies and FcR bloking reagent (Miltenyi, #120-000-442) overnight and washed third in PBS. Slides were mounted with the Fluoromount Kit containing 4-, 6-diamidino-2- phenylindole (Invitrogen) to counterstain the nuclei. The slides were analyzed with a Zeiss confocal microscope Laser Scanning Microscope 710 (Carl Zeiss). Pictures were analyzed with Zen 2008 V5,0,0228 software (Carl Zeiss).

### Cytokine array

Cell culture supernatant was collected and proteins quantified based on Bradford assay. 200 μg of protein was loaded on R&D system® Human Cytokine Antibody Array panel A (R&D system®) according to manufacturer’s instructions. Arrays were revealed using HorseRadish Peroxidase (HRP) and SuperSignal West Pico Chemiluminescent Substrate (Thermo Scientific). Data were collected using Geliance CCD camera (Perkin Elmer), and extracted using ImageJ software (NIH). Briefly pictures were inverted and background subtracted. We defined a120 micron diameter area signal capture for all spots. We defined our signal as the median pixel density value. For comparison, independent array values were normalized on their positive control intensity values.

### Migration and invasion assays

#### Wound healing assay

OCC OVCAR3 (50 000 cells/well) were plated in 24-well plates in triplicate for each condition. The cells were starved from serum during 24 hours in a serum free cytokine free context. The same straight scratch was made in all the wells with a 1 μL pipette tip. After washing the well with PBS to remove the detached cells, a fresh medium containing specific molecule for each condition was used, including positive control 20% serum medium and negative control serum-free, cytokine-free medium. The cytokines IL6 (100 ng/ml, #20006 PeproTech), IL8 (100 ng/ml, #20008 PeproTech), TNFa (100 ng/ml, #AF-30001A PeproTech) and Rantes (100 ng/ml, #30006 PeproTech) were added as indicated. Photos were taken and the rate of closure was determined at 6, 12, 24 and 48 hours. Experiments were performed in triplicate.

#### Migration and invasion assays

Assays were done in invasion chambers pre-coated with reduced growth factor matrix from BD Biosciences. 50 000 viable cells were added to the upper chamber in 200 mL of serum-free medium and incubated in 5% CO2 at 37°C. For invasion assays, the lower chamber was filled with 600 mL of specific medium for each condition: 100 ng/mL IL6, 100 ng/mL IL8, 100 ng/mL Rantes, 100 ng/mL TNFa, positive and negative controls. OCC migration and invasion was then assessed counting the cells at the bottom of the well. Experiments were performed in triplicate.

#### IL6 inhibition

Inhibition of IL6 receptor was performed by incubation of OCC and AMS in ultralow attachment plate (Corning) with specific anti-IL6 receptor antagonist antibody (Abcam, #ab47215) at the concentration of 1 μg/ml in ultralow attachment 48 well plate (Corning) containing DMEM/F12 (1:1) (Hyclone) basal media supplemented with 2 mM L-Glutamine (Hyclone), 1 × Non Essential Amino Acid (NEAA) (Hyclone), PenStrepAmpB (Sigma), 20 ng/ml basic Fibroblast Growth Factor (bFGF) (Peprotech), 20 ng/ml Epidermal Growth Factor (EGF) (Peprotech), 5 μg/ml Insulin (Sigma), 2% B27 supplements (Invitrogen) and 4% basement matrigel (BD Biosciences). A control was performed using an IgG1 isotype antibody. After 24 hours, analysis of the infiltration was performed by confocal microscopy (as described above).

### Statistical analysis

Student-t tests, Fisher exact tests and chi-square tests were performed as appropriate. All p-values are two-sided with statistical significance evaluated at the 0.05 alpha levels. Ninety-five percent confidence intervals (95% CI) were calculated to assess the precision of the obtained estimates.

## Results

### Cancer cell invasion of amniochorionic membrane scaffold (AMS) recapitulates peritoneal metastasis

**Figure 1:**
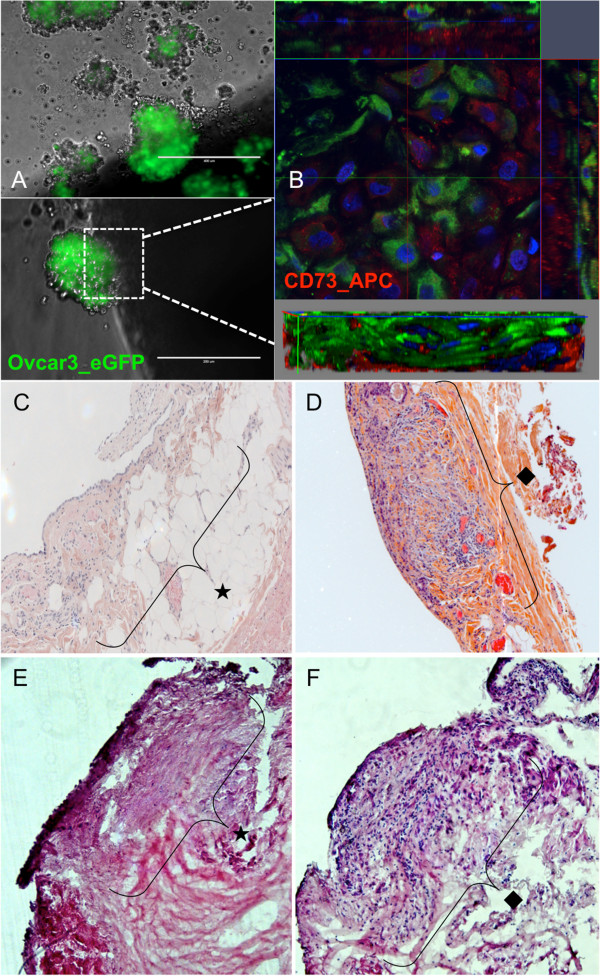
**Amniochorionic membrane based 3D model: involvement of the amniochorionic membrane scaffold (AMS) by ovarian cancer cell lines compared to histologic involvement of the peritoneum. A**. Picture by fluorescence microscopy of Ovcar3_eGFP aggregates after 24 hours 3D culture (upper picture). Early attachement (day 1) of the tumoral aggregates on AMS in fluorescence microscopy (lower picture). **B**. Early involvement of AMS stained with CD73 APC (red) by Ovcar3_eGFP (green) in confocal microscopy. On this Z-stack reconstruction of a small tumor, we could see the pattern of eGFP-OCC infiltration within the AMS. Amniotic stromal cells CD73+ were located in surface but also deeper within AM, surrounding OCCs. **C-F**. On the histologic sections stained with hematoxylin & eosin, the area below the mesothelium (star) of a normal peritoneum (**C**) and amniochorionic membrane (**E**) was free whereas it was filled with tumoral cells (rhombus) in sections of peritoneal metastasis (**D**) and involved AMS at day 2 (**F**).

We cultured OCCs in suspension in serum-free media contining 4% matrigel with small pieces of amniochorionic membrane, referred as amniotic membrane scaffold (AMS). We obtained OCC aggregates within the 24 first hours (Figure 1A). Within the 24 first hours OCC aggregates and AMS culture, we could see some of the aggregates in contact with the AMS. We observed adhesion of eGFP-OVCAR3 and eGFP-SKOV3 aggregates on the AMS with optic and fluorescent microscope after 24 hours (Figure 1A). We could take the AMS with a 100μL tip in a culture dish and wash it with PBS to remove unattached OCC at the surface of the AMS. The AMS was fixed with 4% paraformaldehyde (PFA) during 10 minutes and analysed in confocal microscopy.

Forty-eight hours after adhesion, we could demonstrate infiltration of eGFP-OCC within AMS stromal cells stained with APC-CD73 using confocal microscopy (Figure 1B), using Z-stack reconstruction. We could then study the pattern of OCC infiltration within AMS and their surrounding amniotic stromal cells. Amniotic stromal cells CD73+ were located in surface but also deeper within AM, very close to the OCCs (Figure 1B). These results were obtained with both cell lines v.

In order to confirm the infiltration we performed histologic examination after hematoxylin & eosin staining and compared morphologic aspect of AMS infiltration with peritoneal metastatic nodules (Figure 1C-F). Peritoneal nodules were characterized by an infiltration of the sub-mesothelial region compared to normal peritoneum (Figure 1C and E). Histologic examination of AMS also revealed a sub-epithelial region, infiltrated by OCC 72 hours after co-culture, thus recapitulating peritoneal carcinosis (Figure 1D and F).

We established a non-adherent co-culture system of OCC aggregates and AMS. As we demonstrated AMS based 3D model could replicate early invasion steps of EOC infiltration mimicking peritoneal carcinosis, we went further and used this 3D model to understand the initial steps involved in invasion.

### OCC enrichment in regions with MSC

**Figure 2:**
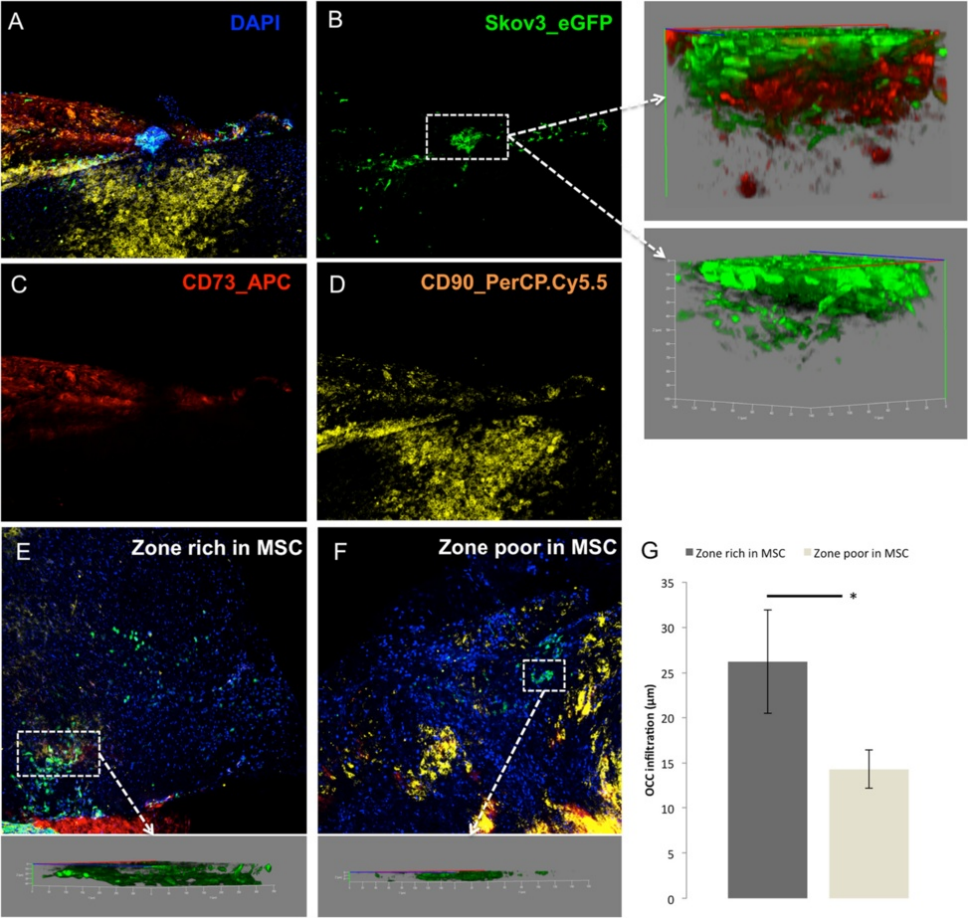
**The distribution of OCC within AMS is following expression of MSC markers. A-D**. Confocal microscopy tile scan reconstruction of Skov3_eGFP (green) distribution after staining with CD90 (red) and CD73 (yellow). The distribution of OCC (**B**) is following the distribution of CD90 (**C**) and CD 73 (**D**). Using 3D z-stack reconstruction of the tumor, we could measure the tumor depth infiltration of OCC within AMS and the relations between OCC and surrounding CD73+ amniotic stromal cells (B, arrows). **E-F**. Confocal tile scan reconstruction showing the distribution of OCC within AMS in regions rich in MSC (**E**) and without MSC (**F**), with their related depth of infiltration. (**G**). OCC infiltration was significantly increased in regions rich in MSC (*p<0.05).

We have previously demonstrated that the AMS has a high content in mesenchymal progenitor cells (defined here by AM CD73^+^CD90^+^ cells) [14]. We have also shown the role of MSCs in resistance to treatment as well as acquisition of a metastatic phenotype [6]. We wondered whether MSCs could have a role in the initial invasion of the AMS. We observed that AMS invasion was not homogenous indeed most of the tumor implants were located in regions rich in MSC (70% tumors per AMS) compared with regions without MSC (30% tumors per AMS) (NS). For this evaluation, we counted randomly 20 OCC nodules within six different membranes, three with eGFP-OVCAR3 and three with eGFP-SKOV3 cells. We found that 6 of the 20 nodules were located in areas rich in MSC markers. The distribution of OCC nodules was correlated with MSC distribution (Figure 2A to 2D). Using 3D z-stack reconstruction, we could measure the tumor depth infiltration of OCC within AMS and the relations between OCC and surrounding amniotic stromal cells (Figure 2B, arrows). We found that tumor infiltration of the AMS was deeper within regions rich in MSC (26.2 +/− 11.2 μm vs 14.3 +/− 4.3 μm, p<0.05) (Figure E to 2G).

We illustrated that MSCs played a role in a 3D model of tumor infiltration of OCC. In this model, the presence of MSC enhanced tumoral aggregates adhesion and infiltration. We investigated potential molecular determinants that could play a role in the cross-talk between OCC and the MSC-niche.

### Interaction between MSC and OCC results in increased migration and invasion through IL6 secretion

**Figure 3:**
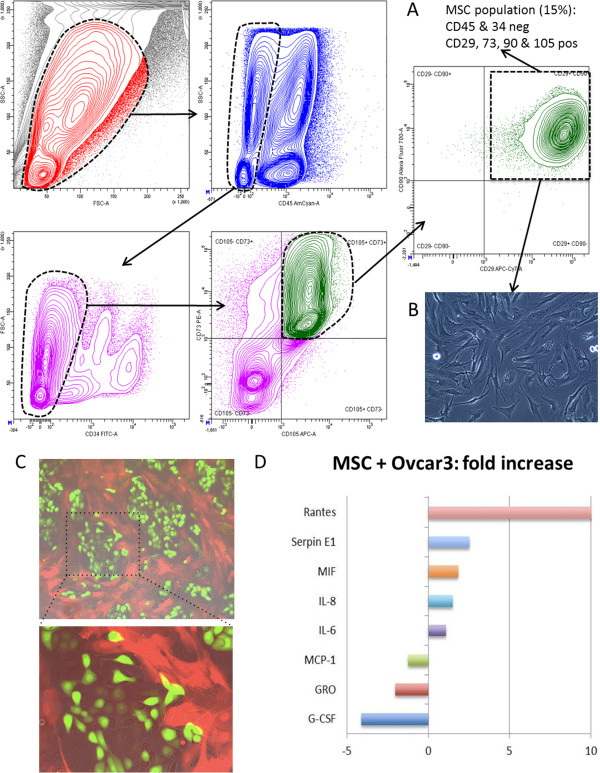
**Cytokine array after extraction and culture of the amniotic MSC with OCC. A**. By flow cytometry analysis, we identified a population of MSC defined by negative for CD45 & 34 and positive for CD29, 73, 90 & 105. This population represented 15% of the total cell population of the amniochorionic membrane. **B**. Picture of the cultured cells after sorting by optical microscopy. **C**. Picture of a coculture between MSC stained with calcein red and OCC (Ovcar3_eGFP in green) by fluorescence microscopy. **D**. Cytokine array results after MSC and OCC coculture.

**Figure 4:**
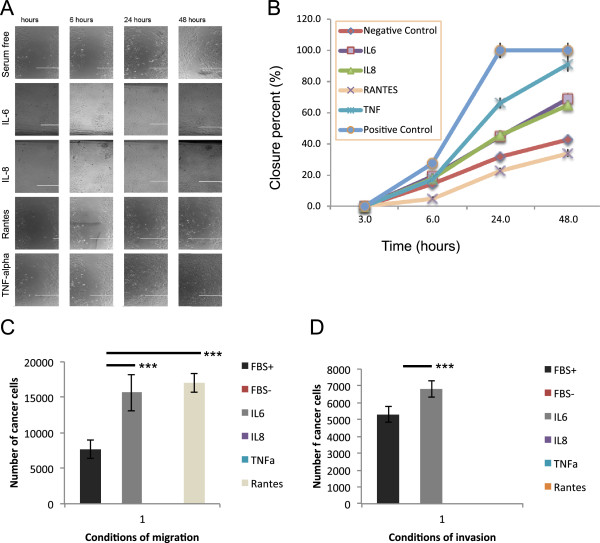
**Wound healing assay and Transwell with Ovcar3 stimulated by IL6, IL8, TNFa and Rantes: A**. Wound healing assay: Pictures of the different conditions at 3, 6 24 and 48 hours. **B**. Wound healing assay: Representation of the rate of closure for each condition. TNFa, IL6 and IL8 significantly increased the migration of Ovcar3 compared with Rantes and serum free medium. The positive control used was complete medium with serum and negative control was serum free medium. **C**. Transwell: Increased migration was observed with TNFa, IL6 and Rantes (p<0.001 ***). No migration was observed in the three conditions FBS-, IL8 and TNFa. **D**. Transwell coated with basement membrane: Increased invasion was observed with IL6 (p<0.001 ***). No invasion was observed in the four conditions FBS-, IL8, TNFa and Rantes.

To study the role of MSC interaction with OCC in vitro, we identified MSC population within the AMS used in this study. As we have previously described, we defined amniotic MSCs as CD90^+^CD73^+^CD105^+^CD29^+^CD45^-^CD34^-^ (Figure 3A), [14]. The MSC population represented 15% of the AMS live cells, as recently reported [14]. They displayed classical mesenchymal phenotype after cell sorting and expansion (Figure 3A-B), concordant with previous data [11]. We established a co-culture system with OCCs. Interestingly, as shown in Figure 3C, OCCs and MSCs formed spontaneously nodular networks where MSC trapped tumor nodules. We hypothesized that secreted mesencrine factors could be responsible for increased invasion; we therefore screened cytokines secreted in a serum-free cytokine-free context in the co-culture setting using cancer cell culture as a control (Figure 3). IL6, IL8 and Rantes had increased secretion in co-culture compared to OCC and MSC monoculture (Figure 3D). Furthermore, TNFa was demonstrated to orchestrate the ovarian tumor microenvironnement and promote tumor progression [15]. While many of the cytokines have been described for their role in cell migration we decided to screen the potential pro-metastatic mesencrine factor using migration (wound healing assay and Boyden chamber), and invasion (Matrigel coated Boyden chamber) assays. In vitro tests revealed that IL6 was the only molecule increasing both migration and invasion. Rantes, IL8 and TNFa increased inconstantly cell migration and invasion (Figure 4). We observed a 1.5 to 15 fold increased migration and >10 fold increased invasion with IL6.

**Figure 5:**
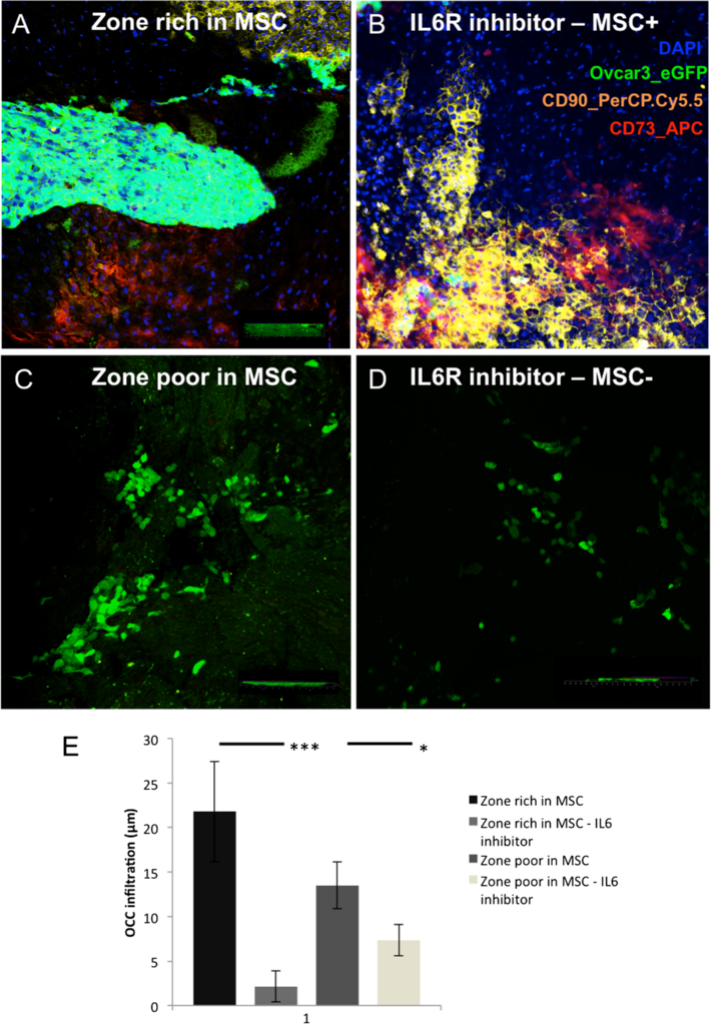
**IL6 receptor (IL6R) inhibition decreased adhesion and infiltration of OCC on AMS. A** and **C**. Confocal tile scan showing distribution of OCC, *i.e.* Ovcar3_eGFP (green), within AMS in regions rich in MSC stained with CD90 (red) and CD73 (yellow) (**A**) and without MSC (**C**). **B** and **D**. Confocal tile scan showing, after IL6R inhibition, distribution of OCC within AMS in regions rich in MSC (**B**) and without MSC (**D**). **E**. OCC infiltration was significantly decreased in regions rich in MSC (p<0.001 ***) and in regions without MSC (p<0.05 *).

The effect of the inhibition of IL6 on AMS infiltration by OCC was tested by the incubation of OCC and AMS in ultralow attachment plate with a specific anti-IL6 receptor (IL6R) antagonist antibody (Figure 5). We observed significant global decreased AMS infiltration when inhibiting IL6R (Figure 5E). The decrease in OCC infiltration was more important in regions rich in MSC, defined as CD73^+^CD90^+^ cells, indicating that recruitment and 3D invasion of OCC was dependent of IL6 secretion (7.2 fold versus 2.1 fold, p=0.03) (Figure 5). The control with IgG1 isotype antibody was performed and showed no effect on OCC infiltration.

## Discussion

In this study we illustrated the important role of MSC in early OCC invasion with a 3D model of metastatic nodule based on an amniochorionic membrane scaffold. We investigated the interaction between OCC and MSC and found that IL6 was determinant for OCC to migrate and infiltrate 3D structures resembling the peritoneum.

Peritoneal mesothelium is the first barrier against spreading ovarian cancer cells. OCC adhere to mesothelial cells via integrins after MMP2-mediated digestion of vitronectin and fibronectin [16]. Previous studies showed that the mesothelium could not be detected under the proliferating OCC implants suggesting that mesothelial cells are dissociated before peritoneal involvement [17,18]. After breaking peritoneal mesothelial layer, OCCs can invade the sub-mesothelial area, constituted by an extra-cellular matrix, and various cell types including MSCs and fibroblasts. Tumoral secreted factors such as leucine, leucine-37 (LL-37) or lysophosphatidic acid (LPA) could recruit MSCs and induce their differentiation in cancer-associated fibroblasts (CAF) through signalling pathways involving Rho kinase, ERK, PLC, and phosphoinositide-3-kinase [19,20]. LPA present in the EOC microenvironment was also reported to induce STAT3 phosphorylation and ovarian cancer cell motility through the secretion of IL-6 and IL-8 [21]. This is in line with the increased invasion of OCCs through the AMS after stimulation with recombinant IL6.

Several authors [22-24] showed that MSCs promoted tumor growth through increased micro-vascularization, stromal networks, and production of tumor stimulating paracrine factors. They also demonstrated that these properties were activated after Mesenchymal Stem Cell Transition to Tumor-Associated Fibroblasts, through the paracrine secretion of IL6. We observed increased production of IL6 among other cytokines in co-cultures of OCCs and MSCs indicating cross-talk between the two cell lines. Mc Lean et al. demonstrated differences between MSCs and tumor associated MSCs [25]. They reported an enhance ovarian cancer stem cell compartment upon the interaction of cancer cells with Tumor associated mesenchymal cells compare to“wild type” MSCs [25]. Similarly Liu et al. [26] reported that MSCs could support breast cancer stem cell compartment through IL6 and IL8 secretion. In concordance we found an increased number of MSCs in most OCCs nodules within the AMS suggesting an important role in the early invasion for the constitution of an inflammatory reactive stroma: “never healing wound theory” [27,28]. As illustrated above the cross-talk between MSCs and cancer cells and the role of mesencrine factors increasing the metastatic potential have been widely illustrated. Using a unique 3D model based on an amniotic membrane scaffold we were able to replicate the early invasion steps, e.g. (i) formation of tumor aggregates, (ii) adhesion to mesothelial layer (iii) microscopic invasion. We illustrated that MSCs could also play a role in very early attachment and invasion of ovarian cancer nodules. Indeed the significant enrichment of MSCs around invasive nodules suggested their ability to provide the adequate signalling cues for attachment and invasion of ovarian cancer aggregates.

IL6 has been associated with progression in multiple cancer types, including ovarian cancer. Increased expression of IL6 and its specific receptor IL6Rα is associated with disease stage [29]. Recent works also reported the importance of IL6 for early metastasic process in EOC. We found that IL6 inhibition limited early adhesion and infiltration of OCCs in our *in-vitro* 3D model. Giridhar et al. showed that IL6 regulated *in-vivo* adhesion of OCCs to the omentum through up-regulation of LY75 [30]. Using mice with conditional IL6Ra deficiency, they found that host IL6 regulation was important for OCC adhesion [30].

In many cancer IL6 has been described as been up-regulated [31]. IL6 signaling occurs through a hexameric complex including specific IL6-alpha receptor (IL6R; glycoprotein gp 80), and a beta-signaling receptor (gp130) [32]. Dimerization of the receptor induces to the activation of Janus Tyrosine Kinase signaling and the translocation of a signal transducer and activator of transcription (STAT) transcription factors [33]. IL6 mainly signals through STAT3 which translocation to the nucleus induces a complex transcriptional program resulting in Inflammation, cell survival, differentiation or prometastatic properties depending the cellular context [34,35].

There are some limitations in our study. We used AM instead of peritoneum because it was uneasy to obtain and ethically difficult to justify regarding the quantity necessary for our study. The large resections of peritoneum mainly occur in patients undergoing debulking surgery for ovarian cancer and therefore could modify inflammation state of the tissue and bias the experiments. Our model also suffers from the fact that we couldn’t affirm by which side OCC invaded the AMS. However, our goal was to investigate the relations between OCC and MSC, and both amniotic and chorionic membrane is rich in MSC in the same proportion. Finally, we didn’t define the origin of IL6 secretion. We showed increased IL6 secretion in co-cultures of OCCs and MSCs but we did not identify if MSCs, OCCs or both secreted it. Spaeth et al. co-injected Skov3 with and without MSCs into mice and demonstrated that MSCs stimulated tumor growth through paracrine production of IL6 [23]. They found MSCs-induced IL-6 secretion to be critical for the enhanced proliferation observed in Skov-3/MSC tumor growth assay [36]. We showed that IL6 stimulation increased OCC mobility and invasiveness and inhibiting IL6 receptor decreased OCCs infiltration in a 3D model. Colomiere et al. reported increased epithelial to mesenchymal transition (EMT) after EGF treatment of OCCs [37]. In their model OCCs secretion of IL6 was increased upon EGF stimulation. Several authors have demonstrated similar findings in other models [38,39]. In our model, increased EMT and/or cancer stem cell compartment upon IL6 stimulation could explain increased invasiveness and this remains to be investigated.

## Conclusions

In conclusion using an amniotic membrane scaffold might allow us to follow early invasion in a 3D context. We were able to demonstrate the essential role of MSCs. Their interactions with OCCs seemed mediated by IL6, which has been described determinant for cancer migration and infiltration mechanisms. Thus cytokine inhibitors interrupting the cross-talk between OCC and MSC such as IL6 should be investigated as a therapeutic approach in ovarian cancer. *In-vitro* 3D models will therefore be useful to screen for potential efficient inhibitors of early invasion.

## Abbreviations

AM: Amniochorionic membrane; AMS: Amniotic membrane scaffold; bFGF: Basic Fibroblast Growth Factor; CAF: Cancer-associated fibroblast; EGF: Epidermal Growth Factor; EOC: Epithelial ovarian cancer; EMT: Epithelial to mesenchymal transition; FACS: Fluorescence Activated Cell Sorting; IL6: Interleukin 6; IL6R: Interleukin 6 receptor; MSC: Mesenchymal stem cell; OCC: Ovarian cancer cell.

## Competing interests

The authors declare that they have no competing interests.

## Authors’ contributions

Conception and design: CT, RL and ART. Acquisition of data: CR, RL, HAL, CR and MW. Analysis and interpretation of the data: CT, RL, EM, MM and ART. Manuscript preparation: CT, RL and ART wrote the manuscript. Manuscript reviewing: CT, RL, MM and ART. All authors read and approved the final manuscript.
